# Mental Well-Being during the COVID-19 Confinement among Adolescents in Catalonia: The Role of Demographic and Other COVID-Related Variables

**DOI:** 10.3390/children9060783

**Published:** 2022-05-26

**Authors:** Cinta Folch, Helena González-Casals, Joan Colom, Marina Bosque-Prous, Tivy Barón-Garcia, Anaís Álvarez-Vargas, Jordi Casabona, Albert Espelt

**Affiliations:** 1 Centre d’Estudis Epidemiològics sobre les Infeccions de Transmissió Sexual i Sida de Catalunya (CEEISCAT), Departament de Salut, Generalitat de Catalunya, 08916 Badalona, Spain; jcasabona@iconcologia.net; 2 Centro de Investigación Biomédica en Red de Epidemiología y Salud Pública (CIBERESP), C/Monforte de Lemos, 3 Pabellón 11, 28029 Madrid, Spain; aespelt@umanresa.cat; 3 Department of Epidemiology and Methodology of Social and Health Sciences, Faculty of Health Sciences of Manresa, Universitat de Vic—Universitat Central de Catalunya (UVic-UCC), Av. Universitària 4-6, 08242 Manresa, Spain; hgonzalez@umanresa.cat (H.G.-C.); sbaron@umanresa.cat (T.B.-G.); aalvarez@umanresa.cat (A.Á.-V.); 4 Sub-Direcció General de Drogodependències, Agència de Salut Pública de Catalunya, 08005 Barcelona, Spain; joan.colom@gencat.cat; 5 Faculty of Health Sciences, Universitat Oberta de Catalunya, Rambla del Poblenou, 156, 08018 Barcelona, Spain; mbosquep@uoc.edu; 6 Departament de Pediatria, Obstetricia i Ginecologia i de Medicina Preventiva, Universitat Autònoma de Barcelona, 08193 Barcelona, Spain; 7 Departament de Psicobiologia i Metodologia en Ciències de la Salut, Universitat Autònoma de Barcelona (UAB), C/de Ca n’Altayó s/n, 08193 Bellaterra, Spain

**Keywords:** adolescents, COVID-19, impact, inequalities, pandemic, prevention, mental wellbeing

## Abstract

This study aimed to describe the impact of the COVID-19 pandemic on the social situation, self-perceived health status, and mental well-being of adolescents in Catalonia during home confinement, and to evaluate factors that are associated with poor overall mental well-being. An online cross-sectional study among a cohort of students (14–18 years old) of central Catalonia (DESKcohort) was performed during June–July 2020. Poisson regression models with robust variance were used to identify variables associated with “poor overall well-being,” measured by the short version of the Warwick–Edinburgh Mental Wellbeing Scale. Out of 303 participants, 42.1% reported a decrease in family income, and 32.8% a loss of parental employment due to the COVID-19 pandemic, and these percentages were higher among people living in low socioeconomic neighborhoods (53.3% and 43.2%, respectively). Overall, 56.8% presented a poor overall well-being. Participants reporting a decrease in their family’s income (aPR = 1.33) and those knowing a close person or family who died of COVID-19 (aPR = 1.42) were more likely to report a poor overall well-being. This study highlights the patterns of inequality and social vulnerability for COVID-19 pandemic outcomes. Considering social inequalities, interventions are needed to mitigate the impact of COVID-19 pandemic on the physical and the psychological wellbeing of children and their families.

## 1. Introduction

At the beginning of March 2020, the World Health Organization (WHO) declared the COVID-19 a global pandemic [1]. Since that moment, the speed of the pandemic spread and the size that it soon reached required urgent measures to face it. Consequently, on the 12 March, according to the European Centre for Disease Prevention and Control recommendations, measures of physical distancing were extended to all of Spain. These included the closure of schools, considering the complications that could have been possibly caused by comorbidities due to seasonal ailments caused by other respiratory viruses, such as influenza virus [2].

The infection by SARS-CoV-2 (severe acute respiratory syndrome coronavirus-2) has been described among all age groups. According to published data, people under 18 have a lower probability of infection [3], and they manifest the derived pathology with less severity than adults [4]. Nevertheless, the impact of the confinement as a public health strategy to decrease the spread of the virus had consequences on the psychological and the physical health of children and adolescents [5]. On the other hand, a study showed that people of 18–25 years had higher levels of stress, anxiety, and depression, in comparison to people aged 26–60 years, because of the COVID-19 confinement. Since the youngest people in this study were mostly university students, the authors concluded that the additional stress experienced by young people may have been due to the new online educational environment, without face-to-face classes [6].

Several factors were negatively associated with the adolescents’ mental well-being, including demographic variables such as female sex [7], changes in household dynamics such as decreased family income [8], and fear of losing family members [9], among others.

This study aimed to describe the self-perceived health status and mental well-being among adolescents of Catalonia during home confinement due to the COVID-19 pandemic and the impact on the economic and on the employment situation of their families. Secondly, the study aims to evaluate potential sociodemographic and other COVID-related risk factors that could make adolescents more likely to experience mental health problems. 

## 2. Materials and Methods

This cross-sectional study was nested in the cohort of young students from central Catalonia (DESKcohort) (http://deskcohort.cat/en/home/ accessed on 23 May 2022). The region of Central Catalonia consists of 150 municipalities with a population density of 88.4 inhabitants per square kilometer. The average annual income per person is lower than the Catalan average. The study population consisted of 14- to 18-year-old students from secondary schools in the academic year 2019–2020 that had participated in the first wave of the DESKcohort survey and agreed to be contacted for further studies. Of these 1583 students, 303 (20%) were surveyed online for this study during June and July 2020. 

We designed an ad-hoc questionnaire that was sent through email or WhatsApp to the contact provided by the DESKcohort participants. It included socio-demographic data such as gender, course, size of municipality, household situation (whether they lived in an apartment or a house, and the presence of a terrace and/or courtyard). We used the Mac Arthur Scale of subjective social status to assess the self-reported socioeconomic level of the neighborhood [10]. To create this variable the following question was asked: “Think of this bar as representing where people stand in society. At the top (100) are the people who are the best off—those who have the most money, the most education and the most respected jobs. At the bottom (0) are the people who are the worst off—who have the least money, least education and the least respected jobs or no job. Where would you place your family on this scale?”. The scores ranged from 100 to 0, and terciles were used to interpret them. A higher score indicated a higher socioeconomic level. This measure has been successfully used with adolescents, and it has a very good test-retest reliability (intraclass correlation = 0.73) [11]. 

Regarding COVID-19 related questions, participants were asked about the impact of the confinement on the employment situation of parents/legal guardians, and on the income of the family. COVID-19 exposure was assessed by asking if they or a relative/close person had been diagnosed with COVID-19, and if this relative/close person had been hospitalized or passed away because of the virus. 

The self-perceived health status during the period of confinement was assessed by the question: “During the confinement, would you say that your health was excellent, very good, good, fair, or poor?” 

To measure overall well-being, the short version of the Warwick–Edinburgh Mental Wellbeing Scale (SWEMWBS) was used. The SWEMWBS uses seven items of the long version questionnaire about thoughts and feelings, which are positively worded with five response categories from “Never” to “Always” during the last two weeks. The seven items were the following: “I’ve been feeling optimistic about the future,” “I’ve been feeling useful,” “I’ve been feeling relaxed,” “I’ve been dealing with problems well,” “I’ve been thinking clearly,” “I’ve been feeling close to other people,” and “I’ve been able to make up my own mind about things”. The SWEMWBS has been validated for populations of young people aged 15–21 [12,13] and the general population [14]. The SWEMWBS was scored by first summing the scores for each of the seven items, which were scored from 1 to 5. The total raw scores were then transformed into metric scores using the SWEMWBS conversion table (which can be found at the following website: https://www.corc.uk.net/outcome-experience-measures/short-warwick-edinburgh-mental-wellbeing-scale-swemws/ accessed on 23 May 2022). Scores ranged from 7 to 35 and higher scores indicated higher positive mental wellbeing. In this study, to interpret the scores, a mean score for the whole sample was estimated. Scores over the mean of the sample were considered as “good overall well-being” whilst scores under the mean were considered “poor overall well-being”.

The data were electronically captured through the REDCap system (Research Electronic Data Capture). The system is installed in the server of the Research and Innovation Department (DRI) [server Fludd] of the Bages University Foundation (FUB) in the Manresa campus of the University of Vic-Central Catalonia University (UVic-UCC) [15]. The project was approved by the ethics committee of the UVic-UCC and the study was conducted according to the guidelines of the Declaration of Helsinki. During the first wave of the DESKcohort project, we generated a personalized code for each student. For this, we used the first two letters of their first name, of their first surname, and of their second surname; and the date of birth of the student. The same code was used to identify the same student in this study. 

The questionnaire was confidential and data treatment followed the Spanish LOPD rules and the corresponding ethical considerations. The families of the participants signed a consent form and students gave explicit consent to take part in the project. 

We performed a descriptive analysis and compared proportions by using a Pearson’s χ^2^ test and a Fisher exact test. Finally, we used Poisson regression models with robust variance to identify potential sociodemographic and other COVID-related variables associated with “poor overall well-being”. Adjusted prevalence ratios (PR) and a respective 95% confidence interval (95% CI) were estimated [16]. We considered a 5% error for all the analysis, and we used the version 20 of SPSS software. 

## 3. Results

A total number of 303 students participated in the study. Boys represented 29.7% of the sample (mean age = 16.3 years) and girls represented 70.3% (mean age = 16.4 years). Of the 303 participants, 169 were attending the last year of compulsory secondary education (equivalent to International Standard Classification of Education (ISCED) 2, according to the United Nations Educational Scientific and Cultural Organization (UNESCO); 104 were attending the last year of the sixth form (Upper Sixth); and 30 the Vocational and Educational Training (VET) courses. The last two courses are equivalent to ISCED 3 [17]. 

**Figure 1:**
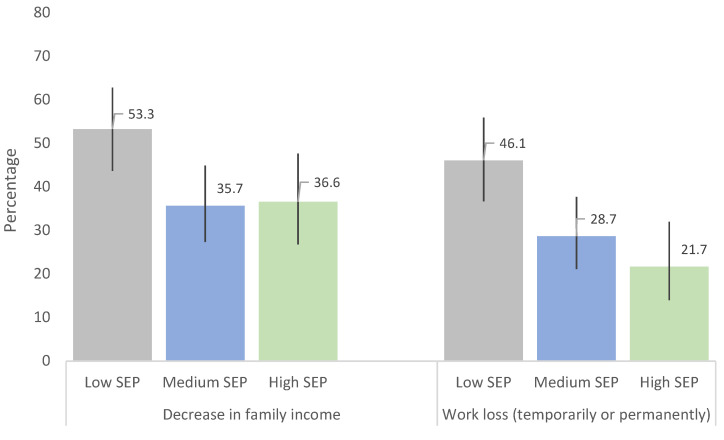
Changes in the economic and in the employment situation during confinement according to the neighborhood socioeconomic position (SEP).

COVID-related variables are presented in Table 1. Most of the participants lived in a flat or a house, and 83.8% had a courtyard or a terrace. A percentage of 42.1% of the participants reported that their family income had decreased because of the pandemic. Moreover, in 32.8% of the cases, there had been a worsening of parents’ employment status (a loss of parental employment temporarily or permanently). These percentages increased for students living in neighborhoods with the lowest socioeconomic level (53.3% and 43.2%, respectively) (Figure 1).

**Figure 2:**
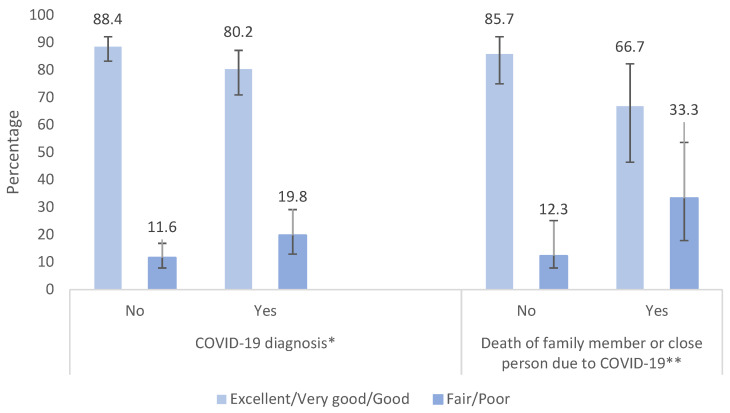
Self-perceived health status according to diagnosis or death of COVID-19 of a close person. * *p* = 0.057, ** *p* = 0.003.

Regarding self-perceived health status during the period of confinement, 85.8% of the sample reported having an excellent, very good, or good self-perceived health status. Adolescents that were diagnosed with COVID-19, or had a family member or close person that was diagnosed with or died of COVID-19 (31.7%, Table 1), had a worse self-perceived health status, in comparison to the rest of the population (*p* = 0.057 and *p* = 0.003, respectively) (Figure 2).

Overall, 56.8% of the participants had a score below the mean on the SWEMWBS, indicating a poor overall well-being. The prevalence of poor overall well-being was higher among girls [61.0% (95% CI: 54.3–67.4)] in comparison with boys [46.7% (95% CI: 36.6–57.0)], people attending the last year of the sixth form (ISCED 3) [61.5% (95% CI: 51.9–70.4)], people attending VET courses [40.0% (95% CI: 24.3–58.1)], people who lived in neighborhoods with a low or a medium socioeconomic level [60.0% (95% CI: 50.3–68.9) and 60.9% (95% CI: 51.6–69.4)] in comparison to people from neighborhoods with high socioeconomic level [47.0% (95% CI: 36.5–57.7)], and those living in a house or flat without terrace or counterpart [75.5% (95% CI: 61.6–85.6)] in comparison with those with these living conditions [53.1% (95% CI: 47.0–59.2)]. On the other hand, participants reporting a fair/poor self-perceived health status had a higher prevalence of poor overall well-being than those with an excellent, very good, or good self-reported health during the confinement [75.7% (95% CI: 59.4–86.9) and 54.1% (95% CI: 48.1–60.1)] (Table 2).

Regarding the COVID-related variables, participants reporting a negative impact of the pandemic on their family economy had a higher prevalence of poor overall well-being than those reporting that their family income had increased or maintained stable during the confinement [67.7% (95% CI: 59.1–75.3) and 48.6% (95% CI: 41.2–56.0)]. The prevalence of poor overall well-being was also higher among participants reporting a loss of parental employment temporarily or permanently due to the pandemic in comparison with those who reported no changes in parental employment [64.6% (95% CI: 54.7–73.4) and 49.2% (95% CI: 40.6–57.9)]. Finally, the proportion of participants reporting poor overall well-being was higher among those who had a family member or close person that was hospitalized [70.2% (95% CI: 55.7–81.5)] or died of COVID-19 [81.5% (95% CI: 62.4–92.1)] in comparison with those who did not report these stressful situations [54.3% (95% CI: 48.1–60.3) and 54.3% (95% CI: 48.4–60.2), respectively] (Table 2).

The results of the univariate and multivariate Poisson regression models with robust variance are presented in Table 3. At univariate level, girls (OR = 1.31; 95% CI: 1.02–1.67) and those self-reporting a fair or poor self-perceived health status (OR = 1.40; 95% CI: 1.13–1.73) were more likely to have a poor overall well-being compared with boys and those reporting an excellent, very good, or good self-perceived health status, respectively. Furthermore, participants with parental job and/or income losses due to the confinement (OR = 1.31; 95% CI: 1.04–1.65 and OR = 1.39; 95% CI: 1.15–1.69, respectively), and those who reported knowing a close person or family member who was hospitalized (OR = 1.29; 95% CI: 1.04–1.61) or died of COVID-19 (OR = 1.50; 95% CI: 1.22–1.85) were reported to have a poor overall well-being. Living in a house with terrace or courtyard was inversely associated with a poor overall well-being (OR = 0.70; 95% CI: 0.58–0.86).

Finally, at multivariate level, participants reporting a decrease in their family’s income (aPR = 1.33; 95% CI: 1.10–1.62) and those knowing a close person or family member who died of COVID-19 (aPR = 1.42; 95% CI: 1.15–1.74) were more likely to report a poor overall well-being. On the contrary, those living in a house with a terrace or a courtyard were inversely associated with a poor overall well-being (aPR = 0.74; 95% CI: 0.61–0.90) (Table 3).

## 4. Discussion

This article describes the experiences of 14- to 18-year-old students of central Catalonia during the first months of the COVID-19 confinement. In particular, we studied the impact of the pandemic on the economic and on the employment situation of their families, and the potential risk factors that could make adolescents more likely to experience mental health problems.

The COVID-19 crisis has had an unprecedented global effect. However, its consequences have been unequally distributed. In this study, a high percentage of teenagers stated a decrease in their family income and/or a parental work stop or loss (41.9 and 32.7%, respectively). The impact was higher on people living in neighborhoods of a low socioeconomic position. Previous pandemics have highlighted inequality patterns, with worse health indicators in most deprived territories [18]. Moreover, a recent study in the city of Barcelona revealed geographic and economic inequalities related to COVID-19. Such inequalities have also been described in other studies [19,20], and they confirm the existence of a social vulnerability that underscores the need for an equity approach to face the pandemic.

The COVID-19 pandemic has also had an impact on the population’s mental health and psychological wellbeing. Several studies showed a relation between quarantine and a high prevalence of anguish symptoms and psychological problems such as anxiety, stress, and depression. Also, quarantine has been associated to emotions such as fear, nervousness, sadness, and guilt [9,21].

In our study, girls self-reported a worse mental health status than boys, although at multivariate level this association was not statistically significant. Emerging evidence indicates that adolescent girls are disproportionately affected by the pandemic compared to adolescent boys. This is in part due to higher levels of pre-existing psychopathology in women as well as gender differences in fear processing and presenting higher levels of fear, tension, and confusion compared to boys during the pandemic [7,22,23,24,25].

A self-reported fair or poor self-perceived health status was also associated with a lower mental well-being at univariate level, a risk factor that has been documented in other populations during COVID-19 [7]. On the other hand, we found that adolescents who knew a close person and/or family member diagnosed with COVID-19, or who died because of it, were at higher risk of presenting a worse self-perceived health status as well as a lower mental well-being. One of the central factors that may generate high levels of stress and anxiety during the pandemic is the fear of COVID-19, and specifically the fear of either being infected, or of infecting loved ones, a factor that may exacerbate pre-existing mental health disorders or elicit extreme anxiety reactions [24].

In addition, lifestyle changes caused by home confinement during the COVID-19 pandemic had a significant psychological impact on the pediatric population [26]. In our study, COVID-19 related household income loss was associated with reporting worse mental well-being among adolescents. Research shows that stressors such as family financial loss adversely affected household dynamics, worsening adolescent mental health [27,28].

Finally, household related variables such as living in a house with a terrace or a courtyard demonstrated a positive influence on the emotional well-being of the participants. These results are in line with research on the importance of housing conditions on people’s health and well-being. The lack of adequate space, terraces, and gardens have contributed to increased stress and aggressiveness, especially among the disadvantaged [29,30].

A first limitation of our study is that the results cannot be extrapolated to the general Catalan teenage population since the sample size is limited and girls are overrepresented. Nonetheless, this is the first study conducted in Spain that describes the impact of the COVID-19 confinement on adolescents. Therefore, our findings may be useful as a starting point to monitor and to evaluate the effects of the pandemic on youngsters. Moreover, mental well-being before the pandemic was not considered, therefore these results must be interpreted with caution due to the lack of pre-COVID-19 data for comparison. Another limitation is that the items regarding adolescent’s wellbeing were asked considering the last two weeks. Therefore, this is a limitation that needs to be considered when we infer the impact of the pandemic over adolescent’s wellbeing. In addition, bias in causal associations inherent to cross-sectional studies cannot be disregarded. Finally, it was not possible to distinguish between a previous diagnosis by the participant or their family member and/or close person, preventing us from assessing the impact of the COVID-19 illness on the mental well-being of the adolescents. 

In conclusion, this study confirms the existence of social inequalities in the impact of the pandemic on the economic and on the employment situation of the adolescent’s families in central Catalonia, and it contributes to the most recent literature on COVID-19 and the impact on adolescents’ health and well-being, which is still scarce in Spain. Our findings indicate that changes in household dynamics due to the COVID-19 pandemic such as decreased family income, and having a diagnosis or a close person/family member who died of COVID-19 were the main risk factors for a lower mental well-being in adolescents. On the contrary, living in a house with outdoor spaces was a protective factor of a poor overall well-being. Moreover, a worse self-perceived health status had been associated with worse mental-wellbeing, stressing the importance of developing preventive programs to mitigate the COVID-19 pandemic impact on both the physical and the psychological wellbeing of children and their families, taking into account social inequalities. Finally, it is essential to conduct more longitudinal studies to analyze the impact of the pandemic on adolescents and to analyze the effects of the COVID-19 crisis on their health, taking into account the social inequalities exacerbated by the pandemic. This information will allow the generation of social and health initiatives to improve their health and psychosocial wellbeing. 

## Figures and Tables

**Table 1:** COVID-related variables.

	*n*	%
Living place during the confinement		
Flat	151	49.8
House	147	48.5
Housing with terrace or courtyard		
No	49	16.2
Yes	254	83.8
Changes in the family’s income due to the confinement		
No, it remained the same	157	52.0
Yes, it increased	18	6.0
Yes, it decreased	127	42.1
Confinement impact on parents/legal guardians employment situation		
No, they work as usual	128	42.4
Yes, they are teleworking	75	24.8
Yes, they have stopped working (temporally)	93	30.8
Yes, they have lost their jobs	6	2.0
COVID-19 diagnosis (of the participant or a family/close person)		
No	207	68.3
Yes	96	31.7
The patient with COVID-19 was hospitalized		
No	256	84.5
Yes	47	15.5
The patient with COVID-19 died		
No	276	91.1
Yes	27	8.9

**Table 2:** Proportion of participants reporting poor overall well-being by socio-demographic, self-perceived health status, and COVID-related variables (*n* = 303).

		*n*	%	CI
Gender	Boys	90	46.7	[36.6–57.0]
	Girls	213	61.0	[54.3–67.4]
Course	ISCED 2	169	56.8	[49.2–64.1]
	Upper Sixth (ISCED 3)	104	61.5	[51.9–70.4]
	VET courses (ISCED 3)	30	40.0	[24.3–58.1]
Socioeconomic position	Low	105	60.0	[50.3–68.9]
	Medium	115	60.9	[51.6–69.4]
	High	83	47.0	[36.5–57.7]
Municipality	Rural	176	55.7	[48.2–62.9]
	Urban	116	57.8	[48.6–66.4]
Self-perceived health status	Excellent, very good or good	266	54.1	[48.1–60.1]
	Fair or poor	37	75.7	[59.4–86.9]
Living place	Flat	151	60.3	[52.2–67.8]
	House	147	53.1	[45.0–61.0]
Housing with terrace or courtyard	No	49	75.5	[61.6–85.6]
	Yes	254	53.1	[47.0–59.2]
Changes in the family’s income	No or increased	175	48.6	[41.2–56.0]
	Decreased	127	67.7	[59.1–75.3]
Changes in parents’ employment situation	No	128	49.2	[40.6–57.9]
	Teleworking	75	58.7	[47.2–69.2]
	Stop working/job lost	99	64.6	[54.7–73.4]
COVID diagnosis (participant/close person/relative)	No	207	53.6	[46.8–60.3]
	Yes	96	63.5	[53.5–72.6]
Close person/relative hospitalized for COVID-19	No	256	54.3	[48.1–60.3]
	Yes	47	70.2	[55.7–81.5]
Close person/relative died for COVID-19	No	276	54.3	[48.4–60.2]
	Yes	27	81.5	[62.4–92.1]

**Table 3:** Risk factors associated with poor overall well-being. Univariate and multivariate Poisson regression models (*n* = 303).

		PR	95% CI	aPR	95% CI
Gender	Boys	1.00			
	Girls	**1.31**	**[1.02–1.67]**		
Course	ISCED 2	1.00			
	Upper Sixth (ISCED 3)	1.08	[0.89–1.32]		
	VET courses (ISCED 3)	0.70	[0.45–1.11]		
Socioeconomic position	Low	1.00			
	Medium	1.01	[0.82–1.26]		
	High	0.78	[0.59–1.03]		
Municipality	Rural	1.00			
	Urban	1.04	[0.85–1.27]		
Self-perceived health status	Excellent, very good or good	1.00			
	Fair or poor	**1.40**	**[1.13–1.73]**		
Living place	Flat	1.00			
	House	0.88	[0.72–1.08]		
Housing with terrace or courtyard	No	1.00		1.00	
	Yes	**0.70**	**[0.58–0.86]**	**0.74**	**[0.61–0.90]**
Changes in the family’s income	No or increased	1.00		1.00	
	Decreased	**1.39**	**[1.15–1.69]**	**1.33**	**[1.10–1.62]**
Changes in parents’ employment situation	No	1.00			
	Teleworking	1.19	[0.92–1.54]		
	Stop working/job lost	**1.31**	**[1.04–1.65]**		
COVID diagnosis (participant/closeperson/relative)	No	1.00			
	Yes	1.18	[0.97–1.44]		
Close person/relative hospitalized for COVID-19	No	1.00			
	Yes	**1.29**	**[1.04–1.61]**		
Close person/relative died for COVID-19	No	1.00		1.00	
	Yes	**1.50**	**[1.22–1.85]**	**1.42**	**[1.15–1.74]**

PR: Prevalence Ratio; aPR: Adjusted Prevalence Ratio; Figures in bold are statistically significant at the 5% level (*p* < 0.05).

## Data Availability

The data presented in this study are available on request through the web: http://deskcohort.cat/en/databases/. Accessed on 23 May 2022.
